# Cigarette smoke preparations, not moist snuff, impair expression of genes involved in immune signaling and cytolytic functions

**DOI:** 10.1038/s41598-019-48822-w

**Published:** 2019-09-16

**Authors:** Gang Liu, Subhashini Arimilli, Evan Savage, G. L. Prasad

**Affiliations:** 1 RAI Services Company, Winston Salem, NC 27105 United States; 2 Eurofins Lancaster Laboratories PSS, Winston Salem, NC 27105 United States; 3 Genome Explorations, Memphis, TN 38132 United States

**Keywords:** Immunosurveillance, Gene regulation in immune cells

## Abstract

Cigarette smoke-induced chronic inflammation is associated with compromised immune responses. To understand how tobacco products impact immune responses, we assessed transcriptomic profiles in peripheral blood mononuclear cells (PBMCs) pretreated with Whole Smoke-Conditioned Medium (WS-CM) or Smokeless Tobacco Extracts (STE), and stimulated with lipopolysaccharide, phorbol myristate and ionomycin (agonists). Gene expression profiles from PBMCs treated with low equi-nicotine units (0.3 μg/mL) of WS-CM and one high dose of STE (100 μg/mL) were similar to those from untreated controls. Cells treated with medium and high doses of WS-CM (1.0 and 3.0 μg/mL) exhibited significantly different gene expression profiles compared to the low WS-CM dose and STE. Pre-treatment with higher doses of WS-CM inhibited the expression of several pro-inflammatory genes (IFNγ, TNFα, and IL-2), while CSF1-R and IL17RA were upregulated. Pre-treatment with high doses of WS-CM abolished agonist-stimulated secretion of IFNγ, TNF and IL-2 proteins. Pathway analyses revealed that higher doses of WS-CM inhibited NF-ĸB signaling, immune cell differentiation and inflammatory responses, and increased apoptotic pathways. Our results show that pre-treatment of PBMCs with higher doses of WS-CM inhibits immune activation and effector cytokine expression and secretion, resulting in a reduced immune response, whereas STE exerted minimal effects.

## Introduction

Chronic cigarette smoking increases risk for the development of cardiovascular disease, stroke, chronic obstructive pulmonary disease (COPD) and cancer^1–4^. The pro-inflammatory milieu induced by smoking is a significant contributor to many of these diseases^5^. In addition, smoking-induced oxidative stress and chronic inflammation alter innate and adaptive immune responses^6^. For example, increases in circulating white blood cell numbers as well as infiltration and activation of macrophages and neutrophils in the pulmonary environment are widely reported in smokers^7,8^. Importantly, humoral and cell-mediated immune responses are also altered with lymphocyte composition and polarization favoring allergic sensitization, and cytolytic and pro-inflammatory functions^9^. Thus, chronic cigarette smoking induces immune dysfunction resulting in immunosuppression, which increases the risk of bacterial and viral respiratory infections and cancer^1,3,10^.

Smokeless tobacco products are a diverse category of oral tobacco products, which include snus and moist snuff. With the acknowledgement of the existence of a risk continuum across different nicotine and tobacco products, non-combustible tobacco products such as moist snuff, are recognized as significantly less harmful than cigarette smoking^11^. The Center for Tobacco Products/U.S. Food and Drug Administration regulates tobacco products through science-based evaluations of their health effects. Hence, the development of a set of functional assays that reflect the diverse pathophysiological effects of cigarette smoking would be useful for rapid evaluation of the comparative effects of different categories of tobacco products.

Cigarette smoking markedly induces inflammation and alters immune signaling pathways^12^. While a substantial body of knowledge has accumulated on the mechanisms underlying smoking-induced chronic inflammation, emerging data suggest that the use of smokeless tobacco products is not associated with such chronic inflammation and altered immune responses. For example, gene expression profiles of peripheral blood mononuclear cells (PBMCs) from non-tobacco consumers were distinct from smokers, but were similar to those of moist snuff consumers^13^. These data suggest that in contrast to moist snuff consumers, smokers experience extensive alterations in inflammation-related pathways.

The oxidative stress-driven pro-inflammatory conditions induced by exposure to combustible tobacco preparations are suggested to be causal in dysregulated inflammation and immune signaling^14^. We and others have utilized PBMCs as an *ex vivo* model test system to assess how different tobacco products may alter the inflammatory status in consumers^14–17^. These studies have shown that combustible, but not non-combusted tobacco product preparations (TPPs) like smokeless tobacco, markedly suppress Toll Like Receptor (TLR) agonist-stimulated cytokine secretion and cytolytic functions of effector cells^14^. The nuclear factor kappa-light-chain-enhancer of activated B cells (NF-ĸB) signaling pathway plays a key role in regulating inflammation and immune responses^18,19^.

To better understand differences induced in PBMCs treated with different TPPs, transcriptomic methods have been utilized. For example, treatment of PBMCs with cigarette smoke condensate resulted in modulation of several genes including SERPINB2, a gene that is activated in response to cell stress^20^. Recently, we showed that treatment of PBMCs with aqueous extracts of cigarette smoke, termed Whole-Smoke Conditioned Medium (WS-CM), induced extensive gene expression differences in a dose-dependent manner^21^. While the treatment with smokeless tobacco extracts (STE) also resulted in notable alterations in gene expression profiles, they were distinct from those induced by WS-CM. In addition, these results demonstrated a down-regulation of many immune modulators following WS-CM treatment.

To gain an understanding of how exposure to tobacco products could impact the responsiveness of immune cells to external challenges, we examined gene expression profiles of TPP-treated PBMCs stimulated with immune activators. Specifically, we performed transcriptional analysis of PBMCs treated with various concentrations of WS-CM or STE followed by stimulation with lipopolysaccharide (LPS), a TLR-4 agonist, in combination with phorbol myristate (PMA)/ionomycin. Our work shows that treatment with cigarette smoke preparations attenuates the ability of PBMCs to respond to immune activation, reducing immune signaling components and effector cytokine secretion. These results contribute to the mechanistic understanding of the increased susceptibility of smokers to microbial infections and cancer. Further, some of the gene expression differences could be further developed into mechanism-based rapid assays that may inform of some aspects of the long-term health effects of tobacco usage.

## Results

### WS-CM pre-treatment affects gene expression in response to immune stimulation

**Figure 1:**
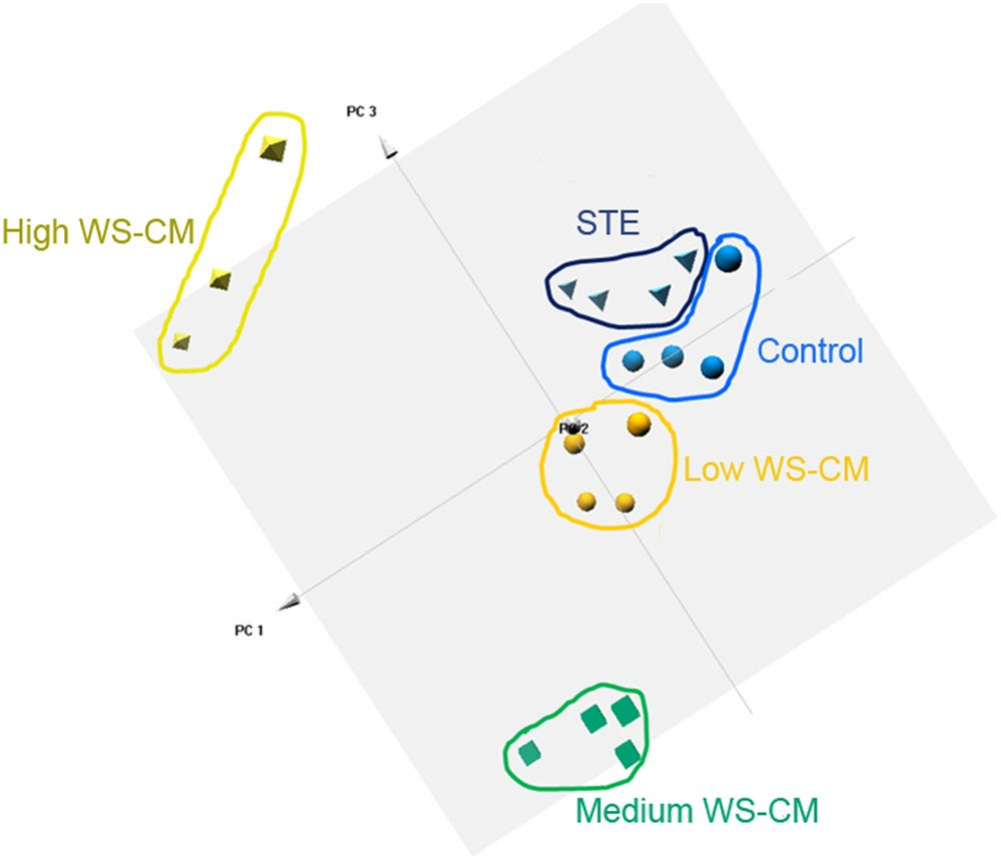
PCA of global gene expression in PBMCs stimulated with PMA, LPS, and ionomycin under equi-nicotine conditions. Human PBMCs were pre-treated with low, medium or high dose WS-CM (0.1, 1.0, and 3.0 µg/mL equi-nicotine units, respectively) or STE (100 µg/mL equi-nicotine units) for three hours followed by stimulation with the agonists (PMA [50 ng/mL], ionomycin [500 ng/mL], and LPS [1 μg/mL]) for five hours. PCA was carried out using R. Gene expression level of all probe sets was dimensionally-reduced and the first top three principal components were presented. While the samples pretreated with high- and medium- WS-CM are distinct and far separated from the control, those pretreated with low WS-CM and STE are more similar and clustered closer to the control samples.

To examine if pre-treatment of PBMCs with STE or different doses of WS-CM alter global gene expression patterns in response to the agonist stimulation, we performed principal component analysis (PCA) on the expression levels for all probe sets on the microarray (Fig. 1). A plot of the samples based on the top three principal components showed that all samples within a treatment group clustered tightly, while their overall gene expression pattern was distinct from the other treatments. Moreover, the control samples, STE pre-treated samples and low dose WS-CM pre-treated samples were clustered closer to each other than to the medium or high dose WS-CM pre-treated samples (Fig. 1). These results show that while the treatments resulted in distinct gene expression profiles, the gene expression changes produced by STE and low dose WS-CM were more similar to control samples than to samples treated with medium or high dose WS-CM.

To compare the effects of STE and WS-CM pre-treatment on the  stimulation-induced gene expression, we performed pairwise analysis of variance (ANOVA) with Benjamini-Hochberg false discovery rate (FDR) adjustment (adjusted p-value < 0.01) for each treatment group compared to untreated controls. We found that pre-treatment with STE and low dose WS-CM did not significantly affect the transcript levels of any genes that were induced by stimulation (Table 1). In contrast, pre-treatment with medium and high dose WS-CM significantly altered the expression of 4808 and 4439 stimulation-induced probesets, respectively (Table 1) Probesets exhibiting differential expression of log2 fold change (FC) >1 and the adjusted p value < 0.01 with medium and high doses of WS-CM are listed in Supplementary Tables S1 and S2, respectively.

**Table 1:** Summary of Differential Gene Expression.

Pair-wise Comparison	Total	>2FC up	>2FC down	>5FC up	>5FC down
Control vs STE	0	0	0	0	0
Control vs low WS-CM	0	0	0	0	0
Control vs medium WS-CM	4808	835	1358	74	186
Control vs high WS-CM	4439	904	1472	157	329

Significantly differentially expressed genes at different fold changes (FDR < 0.01).

**Figure 2:**
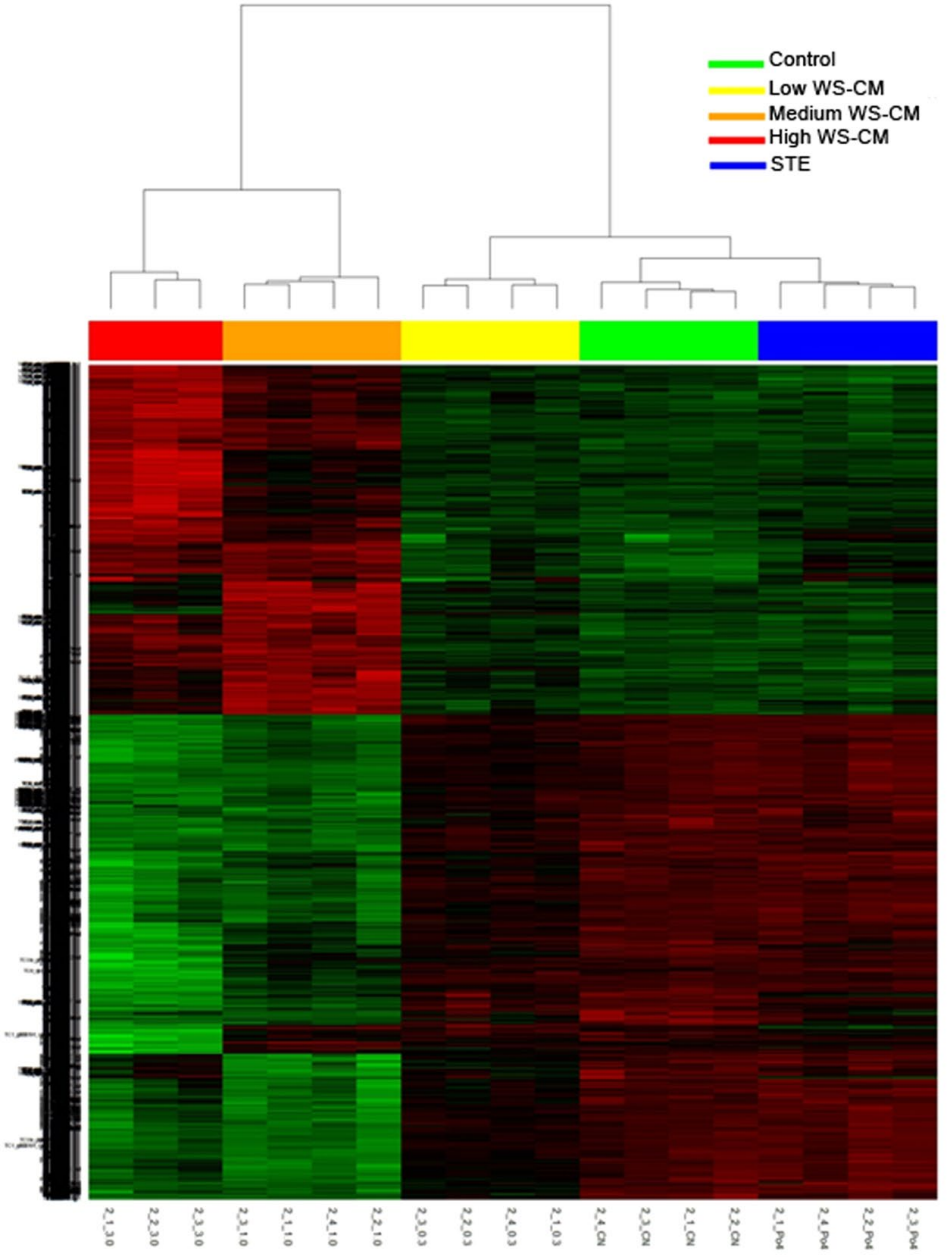
Hierarchical clustering of differentially expressed genes (DEGs) in stimulated PBMCs pretreated with WS-CM or STE. DEGs (<2-fold change in down-regulated or >2-fold change in up-regulated genes; *P* < 0.05) were identified by analyzing the CEL files in R.

Using the combination of all probesets for which expression levels were significantly altered (absolute log2 FC >1) by medium and high dose WS-CM (Supplementary Fig. S1), we performed hierarchical clustering (Fig. 2). Consistent with the PCA results above, the expression profile of low dose WS-CM samples was similar to the profiles of STE and media control samples. In contrast, the gene expression profiles of medium and high dose WS-CM were similar to one another and distinct from the low dose WS-CM, STE, and media control samples. The medium and high doses of WS-CM, compared to the control, induced a large number (2913 and 2376, respectively) of differentially expressed genes (DEGs). While a larger number of the DEGs were distinct between the treatments, several genes were common and concordantly regulated. A total of 278 genes were commonly upregulated and 806 genes were commonly downregulated between the two treatments (Supplementary Fig. S1).

**Figure 3:**
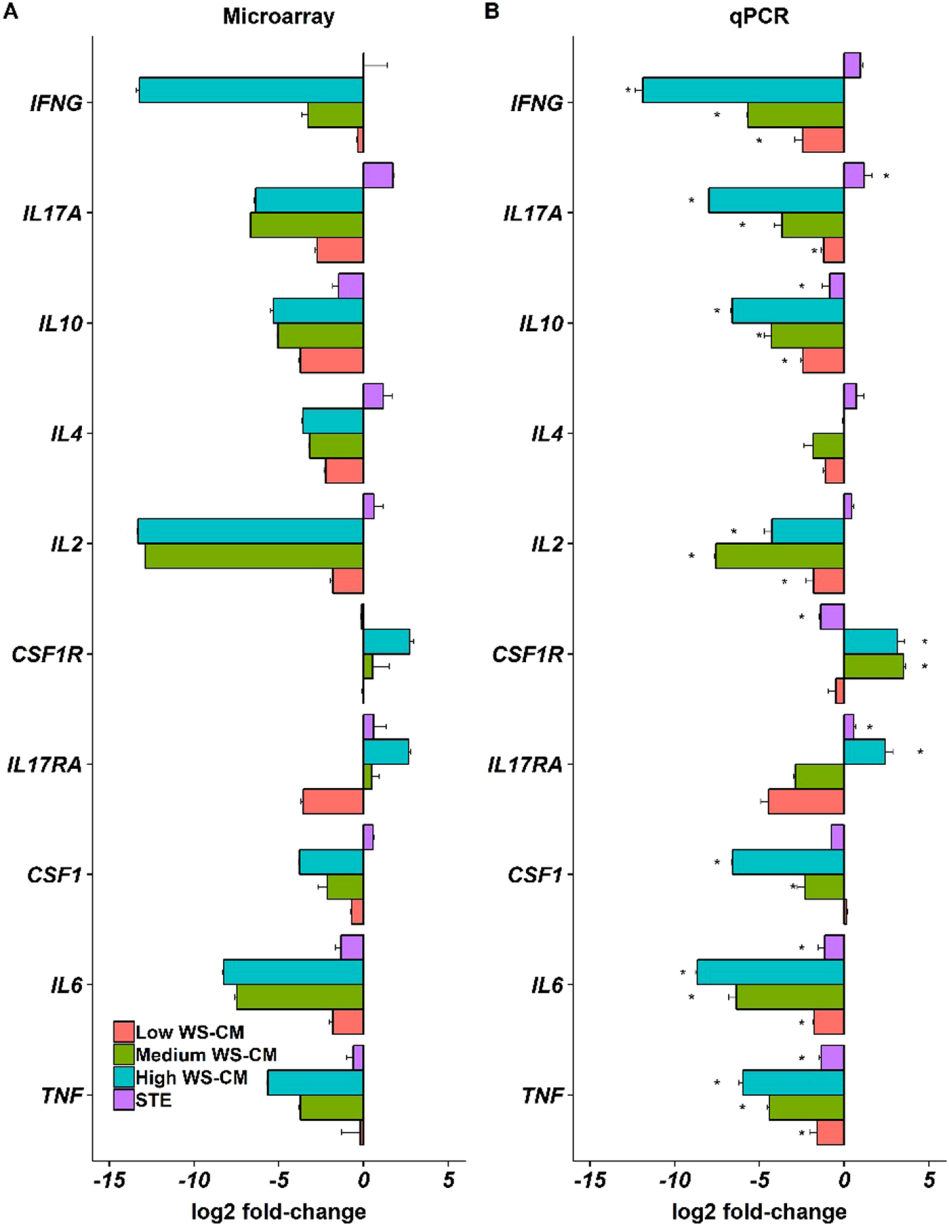
Confirmation of differential gene expression by RT-qPCR. A set of differentially expressed genes from the microarray profiling was analyzed by RT-qPCR as described in the Materials and Methods. The RT-qPCR data (Panel B) confirm the differential gene expression profiling (Panel A).

To validate the expression changes observed by microarray analysis, we performed quantitative RT-PCR on a subset of 10 cytokine and cytokine receptor genes (macrophage colony-stimulating factor 1 [*CSF*-*1*], *CSF*-*1R*, interferon gamma [*IFN*-*γ*], interleukin [*IL*]-*10*, *IL*-*17A*, *IL*-*17RA*, *IL*-*2*, *IL*-*4*, *IL*-*6*, and tumor necrosis factor alpha [*TNF*-*α*]). PBMCs for this assessment were from the same donor samples used for microarray profiling. Transcript levels were normalized to four different housekeeping genes (beta-2-microglobin [*β2M*], *β*-*actin*, Peptidylprolyl Isomerase A [*PPIA*], and TATA-box binding protein [*TBP*]). The results of the expression changes for microarray and RT-qPCR are shown in Fig. 3.

In general, the expression changes were highly correlated between the two platforms (average Pearson Correlation 0.904). The individual correlation between microarray and RT-qPCR for each gene across the various treatment groups is shown in Supplementary Table S3. Both the RT-qPCR and microarray results demonstrate that pre-treatment with low dose WS-CM and STE did not significantly alter the induction of cytokine expression induced by agonist stimulation, while pre-treatment with medium and high dose WS-CM was suppressive for most cytokines. More specifically, lymphocyte specific cytokine transcripts (IL-4, IL-2, IL-17, IL-10 and IFNγ) were induced to a similar level as control samples with STE treatment, but these transcripts were reduced in a dose dependent manner with WS-CM exposure. Transcript levels for pro-inflammatory cytokines (TNF-α, IL-6 and CSF-1) were markedly suppressed by pretreatment with medium and high dose WS-CM; the reduction in the degree of induction of TNF-α and IL6 cytokine transcripts was modest when cells were treated with low dose WS-CM or STE. Interestingly, CSF-1R was induced with stimulation in PBMCs that were pretreated with medium and high dose WS-CM, while IL-17RA was induced above control with high dose WS-CM.

### WS-CM pre-treatment inhibits immune activation

**Figure 4:**
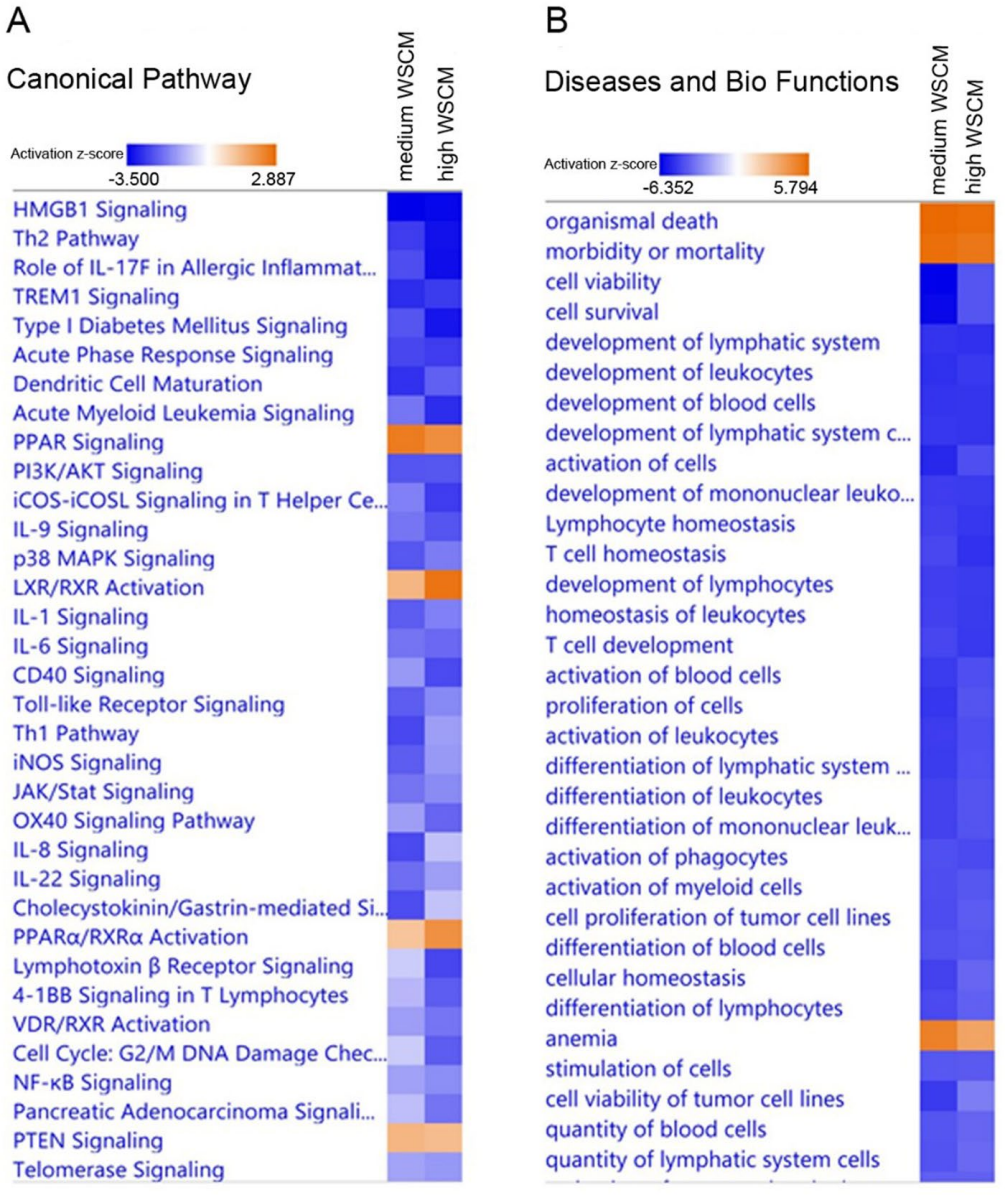
IPA heatmap. Heatmap showing Ingenuity Pathway Analysis (IPA) canonical immune pathways altered by WS-CM or STE treatment and significantly affected by stimulation with PMA, ionomycin, and LPS. Pathways are ranked according to the z-score that predicts activation (orange)/suppression (blue).

To investigate the functional changes produced by higher doses of WS-CM treatments, we used the Ingenuity Pathway Analysis tool (IPA, Qiagen) to identify enriched canonical pathways, diseases and biological functions, and upstream regulators (Fig. 4). Consistent with our previous study^21^ and those from other groups^22^, we found that the canonical Th2 pathway is suppressed by both medium dose WS-CM (z-score = −2.668) and high dose WS-CM (z-score = −3.273) treatments (Supplementary Table S4). Network analysis of the TLR signaling pathway revealed that the expression levels of Interleukin-1 receptor associated kinase 2 (IRAK2) (−10.0 fold), TNF receptor associated factor 1 (TRAF1) (−12.5 fold), NF-κB Inhibitor Alpha (−3.8 fold) and NF-kB1 (−12.5 fold) are reduced, while cFOS (2.4 fold) and CD14 (14.89 fold) expression were upregulated by pre-treatment with higher doses of WS-CM (Supplementary Table S2). Upstream regulator prediction analysis also showed suppression of the LPS (z-score = −7.901 and −7.789 by medium and high dose WS-CM, respectively) and TLR4 (z-score = −5.195 and −5.685 by medium and high dose WS-CM, respectively) regulatory pathways (Supplementary Table S5). These results indicate that equi-nicotine 1.0 and 3.0 ug/mL doses of WS-CM reduced the capacity of PBMCs to respond to a TLR agonist.

In addition to diminishing Th2 signaling, WS-CM significantly inhibited the expression of a wide range of cytokine signaling pathways such as IL-1 (z-score = −2.236 and −1.732 by medium and high dose WS-CM, respectively), IL-6 (z-score = −1.897and −2.064 by medium and high dose WS-CM, respectively), IL-17A (z-score = −0.817 and −1.667 by medium and high dose WS-CM, respectively), and IL-8 (z-score = −2.496 and −0.853 by medium and high dose WS-CM, respectively) signaling (Supplementary Table S4). Upstream regulatory analysis also predicts suppression of TNF-α, IL-2, and IFNγ signaling pathways. Consistent with these results, the major biological functions affected include activation of leukocytes (z-score = −5.022 and −4.392 by medium and high dose WS-CM, respectively) and suppression of phagocytes (z-score = −4.360 and −4.527 by medium and high dose WS-CM, respectively) (Supplementary Table S6).

WS-CM treatment also was  associated with enhanced activity of some cellular pathways. For example, organismal death was increased by WS-CM (z-score = 5.715 and 5.631 by medium and high dose WS-CM, respectively) (Supplementary Table S6). In addition, enhanced signaling was observed in the cell survival-associated peroxisome proliferator activated receptor (PPAR) network (z-score = 2.530 and 2.183 by medium and high WS-CM, respectively) (Supplementary Table S4).

### WS-CM inhibits the expression and secretion of effector cytokines

**Figure 5:**
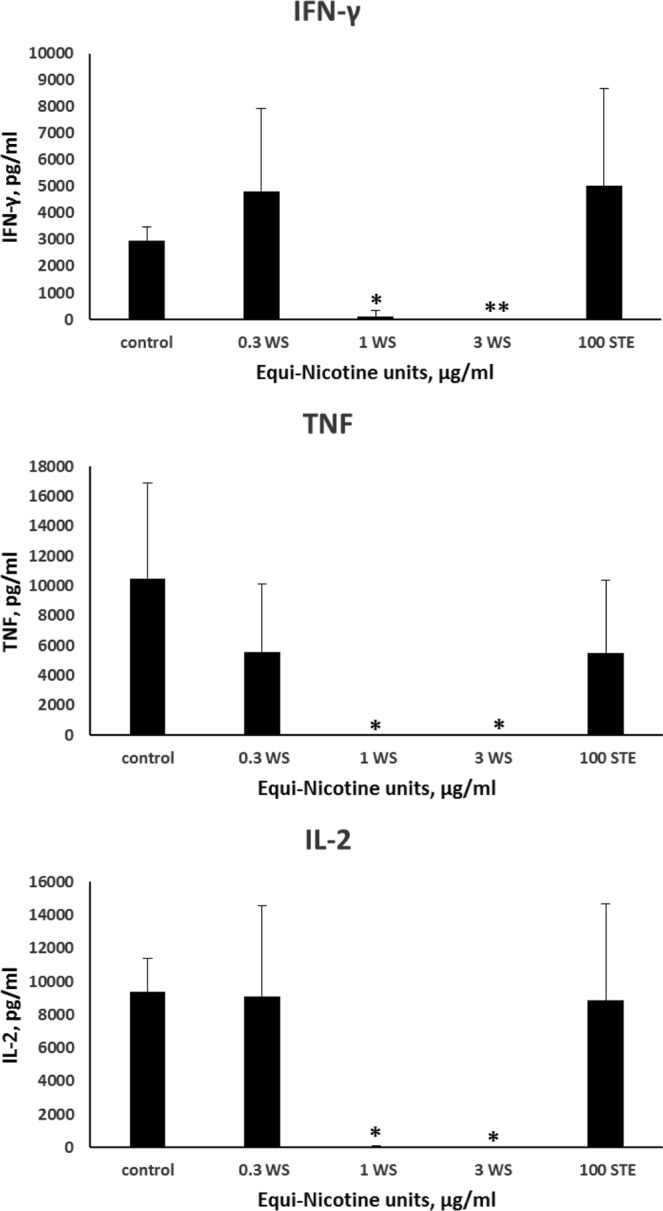
Cytokine expression. PBMCs were pretreated with different concentrations of WS-CM or 100 µg/mL equi-nicotine units of STE for 3 hours and stimulated with the agonists for 24 hours. Levels of cytokines in the culture supernatants were determined using a cytometric bead array and flow cytometer. Each point represents the mean ± SD error bars of four donors from a representative experiment. Since the 3 cytokine levels with high dose treatment and the TNF levels in medium dose WS-CM were below the limits of detection, lowest detectable values were used to compute statistical significance. The statistical significance was indicated by: *P < 0.05 and **P < 0.005.

Based on the pathway analyses and upstream regulator predictions, there appears to be  a loss of signaling capacity of NF-ĸβ and also decreased expression of TNF, IL-1β, IL-2 and IFN-γ with WS-CM pretreatment^21^. We therefore investigated whether the WS-CM treatment resulted in the reduction of secretion of TNF, IFN-γ and IL-2 from stimulated PBMCs. For these experiments, PBMCs were treated with STE or various doses of WS-CM for 3 hours, followed by 24 hour stimulation with the agonists (Fig. 5). Stimulation of the untreated PBMCs with the agonists resulted in a robust upregulation of IFN-γ (2955 ± 511 pg/mL), TNF (10487 ± 6436 pg/mL) and IL-2 (9370 ± 2017). Pre-treatment of PBMCs with low dose of WS-CM prior to stimulation did not significantly alter the cytokine secretion (IFN-γ, 4828 ± 3098 pg/mL; TNF, 5570 ± 4561 pg/mL, and; IL-2, 9106 ± 5483 pg/mL). Pretreatment with medium WS-CM profoundly suppressed IFN-γ (118 ± 205 pg/mL), TNF (below detection limit), and IL-2 (36 ± 62 pg/mL) secretions (Fig. 5). Pretreatment with high dose of WS-CM resulted in a complete suppression of all three cytokines and the levels were below the detection limit. The STE treatment did not significantly reduce the IFN-γ (5016 ± 3656 pg/mL), TNF (5504 ± 4859 pg/mL) and IL-2 (8844 ± 5816 pg/mL) secretions.

## Discussion

Chronic inflammation due to cigarette smoking is a multifaceted process ranging from the recruitment and activation of inflammatory cells to immunosuppression. A key objective of our research is to gain insight into the mechanisms by which chronic cigarette smoking alters the immune response. The objective of this study was to evaluate the impact of WS-CM and STE on global gene expression profiles in PBMCs following activation with widely utilized signal transducers. A key finding from this study was that medium and high dose WS-CM treatment led to marked changes in gene expression profiles and secretion of effector cytokines including TNF, IFN-γ, and IL-2. In contrast, STE pre-treatment did not have a significant effect on gene expression and cytokine secretion of PBMCs. Together, these data demonstrate that, unlike STE, WS-CM dose-dependently diminishes the agonist-stimulated immune signaling in PBMCs. Further, our data show that combustible tobacco products are potent suppressors of the immune response.

Our previous work using an *ex vivo* PBMC model exposed to various TPPs, including WS-CM and STE, demonstrated that exposure to combustible and non-combustible tobacco products resulted in distinct transcriptional profiles, with down-regulation of various immune modulators including TLR4, NF-ĸβ and IRAK2 when treated with high doses of WS-CM^21^. The goal of the current study was to determine whether pretreatment with the TPPs differentially modulates the downstream activation and response to immune stimuli. PBMCs from healthy, non-tobacco consuming donors were treated with various TPPs and then stimulated with LPS, a TLR4 agonist, in addition to PMA and ionomycin, to broadly activate signaling pathways in PBMCs.

Here we show that combustible and non-combustible TPPs result in distinct gene expression profiles after immune stimulation. Pretreatment with STE or low dose WS-CM did not alter stimulus-induced transcriptional profiles. Pretreatment with medium- or high dose WS-CM, however, resulted in transcriptional profiles that were distinct from control (and also STE and low dose WS-CM) and from each other (Figs 1 and 2). A significant number of genes were common between the medium and high dose WS-CM treatment groups and were concordantly regulated (Fig. S1). The microarray and qPCR data demonstrate that agonist-induced effector cytokine secretion was reduced with exposure to higher doses of WS-CM, as evidenced by a lack of *IFNG*, *IL17A*, *IL10*, *IL4*, *IL2*, *CSF1*, *IL6 and TNF* gene induction. Low dose WS-CM and STE did not have a similar inhibitory effect (Fig. 3) on these genes. This result is consistent with the reduction in gene expression and protein production of pro-inflammatory cytokines (TNF-α, IL-1β and IL-6) in alveolar macrophages isolated from non-smokers and smokers and subsequently stimulated *in vitro*^21,23^. The pro-inflammatory molecules IL-1β, TNF-α, IL-2 and IFN-γ are known to function in NK cell and cytotoxic T cell activation which is required to eliminate transformed cells^24^. Thus, they are essential in host defense against infection and cancer.

Analysis of canonical pathways based on expression profiles following immune system stimulation demonstrate that medium and high dose WS-CM exposure similarly alter signaling pathways with inhibition of many pathways involved in immune activation: NF-ĸB, IL-8, IL-6, Th1, Toll like receptor, triggering receptor expressed on myeloid cells 1 (TREM1), Th2 and high mobility group box 1 (HMGB1) signaling (Fig. 4A). The transcription factor NF-ĸβ is a master regulator of immune activation in both innate and adaptive immune cells and it integrates signals from numerous pathways, including TLR4 and the T cell receptor. Mechanistically, the NF-κB signaling is a key driver of cigarette smoke-induced inflammation. Multiple lines of evidence point to upregulation of NF-κB pathway in smokers as well as in smoking-related diseases such as COPD^12^. As many of the gene products shown to be altered in this study are targets of NF-ĸβ, the data suggest that exposure to high doses of WS-CM results in a lack of induction of this signaling pathway, which in turn results in muted activation of NF-ĸβ-regulated pathways such as IFN-γ,IL-2, IL-6, IL-8, IL-9, IL-17, IL-10, TNF-α and CD40 signaling.

TREM1, which belongs to the immunoglobulin superfamily of cell surface receptors, is activated by its putative ligand HMGB1^25^. Functionally, TREM1 is reported to augment immune cell responses together with TLR4, although it is known to inhibit immune signaling under some conditions. Circulating levels of TREM1 (or its soluble forms) have been reported to be higher in different inflammation-related diseases and are proposed as potential biomarkers. Consistent with the downregulation of immune responses, in medium and high doses of WS-CM treated PBMCs, TREM1 signaling is predicted to be downregulated (Fig. 4A).

Additionally, we find that the HMGB1 signaling pathway is down-regulated in cells pretreated with medium and high dose WS-CM (Fig. 4A). HMGB1, a highly conserved non-histone chromosomal protein, is passively released by cells undergoing unscheduled death (necrosis), but not apoptosis^26^. HMGB1 is released in response to cigarette smoke-induced cellular damage, and is reported to mediate inflammatory signaling through TLR4 dependent pathways^27^.

Another signaling pathway involved in NF-kB mediated immune function that is down-regulated by the exposure of higher doses of combustible TPP, but not STE, is the PI3K-Akt pathway. This pathway is critical for cellular survival and activation, as well as immune cell proliferation. Signaling through Akt results in NF-ĸB activation which in turn down-regulates the phosphatase and tensin homolog (PTEN). PTEN is a negative regulator of the Akt pathway^28^, consistent with the increased expression of PTEN seen in the current study (Fig. 4A).

Very few canonical signaling pathways were increased in the current study. Peroxisome proliferator-activated receptor (PPAR) and LXR/RXR signaling pathways showed the greatest increase in activity with exposure to the combustible TPPs. PPARs are members of the nuclear hormone receptor family that act as heterodimers with 9-*cis*-retinoid acid receptors to regulate metabolic pathways as well cell differentiation^29^. Consistent with our data, PPAR ligands, specifically ligands for PPARα and PPARγ, inhibit the activation of inflammatory gene expression and negatively interferes with the inflammatory response^30^. In addition, PPARα has been shown to decrease TLR4 levels and inhibit NF-κB signaling pathway through suppression of TLR4 transcriptional activity^31^.

In our previous study, we demonstrated that exposure to the high dose WS-CM down-regulated mRNA levels of TLR4, IRAK2 and NF-ĸB, key players in the immune response^21^. The current study confirms these results in that effector molecules related to the NF-ĸβ pathway are not increased in response to an immune stimulus when cells are pretreated with higher doses of combustible tobacco products. These results suggest that combustible products, but not STE, exert potent immune suppression due to defective signaling through the NF-ĸβ-signaling axis. Similar to our findings, exposure to cigarette smoke has also been shown to inhibit NF-ĸβ signaling and activation of pulmonary defense mechanisms in response to bacterial infections^19^.

The *ex vivo* exposure study is designed to evaluate the short-term dose-response effects on immune signaling which could inform some of the long-term effects of tobacco use on immune responses. A major limitation is that we used PBMCs from non-tobacco consumers and evaluated signaling and gene expression responses under *ex vivo* conditions, rather than utilizing PBMCs from long-term tobacco consumers. Previously we have reported on gene expression in PBMCs isolated from chronic smokers and moist snuff consumers, and short-term *ex vivo* exposures. Concordant expression was noted in several genes between PBMCs isolated from chronic tobacco users^13^, and those from non-tobacco users and treated with TPPs under the *ex vivo* conditions^21^, and thus the *ex vivo* gene expression data are useful in informing the effect of tobacco use in consumers.

In summary, treatment with medium and high dose combustible TPPs profoundly alters gene expression profiles in the agonist-stimulate PBMCs, which point to an immune-suppressive environment found in chronic inflammation. This key finding is in agreement with the known immunocompromised state of generally healthy smokers, which increases their risk for microbial infections, COPD and cancer.

## Methods

### Tobacco product preparations (TPPs)

WS-CM was generated from reference cigarettes under ISO smoking conditions as described previously^32^. Briefly WS-CM was prepared by passing smoke from four 3R4F reference cigarettes through Roswell Park Memorial Institute (RPMI) 1640 medium without phenol red using the following smoking regimen: 35-60-2, puff volume in mL, puff interval in seconds, and puff duration in seconds, respectively. The pH of the WS-CM stock solution was 7.47 and did not significantly differ with that of the RPMI medium (pH 7.5). STE was prepared by extracting 2S3 smokeless tobacco reference product (North Carolina State University Tobacco Services Analytical Laboratory) in phosphate buffered saline (PBS) for 2 hours followed by filtration. Both TPPs were aliquoted and immediately stored at −80 °C. Aliquots of WS-CM and STE/PBS were analyzed to determine the levels of nicotine, and the dosing was normalized to the nicotine content, which was expressed as equi-nicotine units (µg/mL) across all treatments^33^. TPPs were classified according to equi-nicotine units as either low dose WS-CM [0.3 µg/mL], medium dose WS-CM [1.0 µg/mL], high dose WS-CM [3.0 µg/mL], or STE (100 µg/mL).

### Cell culture and treatment

The clinical study from which samples were obtained was approved by Schulman Associates IRB, Sunrise, FL (now part of Advarra, Columbia, MD) and all subjects provided written informed consent prior to any study procedures being performed. All study procedures were conducted in accordance with applicable sections of the US Code of Federal Regulations (21 CFR 50, 54, 56) and the International Conference on Harmonisation Guidance on Good Clinical Practice.

Whole blood was collected from four healthy non-tobacco user donors at Clinical Contract Research Organization (High Point Clinical Trials Center, High Point, NC). PBMCs were then isolated from whole blood as previously described and immediately frozen and stored in a cryofreezer under liquid nitrogen^32^. PBMCs (3 × 10^6^; n = 3–4 individual donors per treatment condition) were cultured in 96 well plates containing RPMI complete medium (RPMI-CM) supplemented with l-glutamine, penicillin/streptomycin, and fetal bovine serum (10%). PBMCs were treated for three hours with either media (control), low dose WS-CM, medium dose WS-CM, high dose WS-CM, or STE. Given that WS-CM is toxic, the equi-nicotine doses of WS-CM used for treating PBMCs herein are very low, ranging from 0.3 μg/mL to 3.0 μg/mL). Under the experimental conditions, the WS-CM is non-cytotoxic^34^. The STE is minimally cytotoxic and we have used a high (100 μg/mL) in this study.

Cawood *et al*.^35^ propose that if a person smoked 20 cigarettes per a day, then each milliliter of blood would contain 0.0033 (20 cig in 6000 ml blood per person per day) cigarette’s worth of smoke-derived constituents. Current WS-CM was prepared as a stock by smoking four 3R4F cigarettes and bubbling through 20 ml RPMI serum free medium; hence, each milliliter of WS-CM contains cigarette-smoke-derived constituents at the level of 0.2 cig/mL (4/20). Such a stock WS-CM preparation may be considered to contain 60 times (0.2/0.0033) the amount constituents in comparison with the estimated blood concentrations in smokers. Thus, the 3.75% WS-CM used in this study is estimated at about 2.25 cigarettes smoked/day (60 × 3.75%), while the 7.5% and 37.5% WS-CM exposures may correspond to 4.5 and 22.5 cigarettes smoked/day respectively.

Following TPP exposure, PBMCs were washed once with RPMI-CM and then stimulated with RPMI-CM (200 µL) containing agonists [phorbol 12-myristate 3-acetate (PMA, 50 ng/mL), ionomycin (500 ng/mL), and lipopolysaccharide (LPS, 1 μg/mL)]. After five hours, PBMCs were collected, lysed, and transferred to RNALater (−80 °C)^21^. It should be noted that under the experimental conditions, WS-CM^34^, or the agonists (unpublished data) do not cause cytotoxicity. Multiple cell types including monocytes and lymphocytes respond to the agonists. Thus, we expect to detect a cumulative response to the stimulation of multiple cell types, as a diverse set of immune cells are expected to be encounter and respond to tobacco use.

### Microarray experiments

RNA isolation and microarray experiments were performed at Genome Explorations (Memphis, TN). Briefly, single stranded cDNA (ss-cDNA, 5.5 µg) was fragmented, biotin labeled, and hybridized to Affymetrix HTA 2.0 GeneChip arrays. Arrays were washed and stained using Affymetrix® fluidics stations and scanned using GeneChip® Scanner 3000. All data analysis was performed using the Bioconductor package in R. Gene expression levels were normalized using the Robust Multichip Average algorithm (RMA). The raw CEL files and normalized gene expression levels were deposited to the NCBI Gene Expression Omnibus (GEO) with accession number GSE127977.

Two-tailed statistical analysis was performed using pair-wise ANOVA, corrected for type-I error using the Benjamini-Hochberg FDR adjustment method. Analysis of variance was used to compare, low, medium, and high WS-CM groups and STE to control samples. Adjusted <0.01 p value and absolute log fold change greater than 1 “to” fold change great than 2 or less than 0.5 were established as the criteria for differential expression.

Hierarchical clustering was performed using union of the differentially expressed genes obtained in pairwise comparisons (i.e., control vs low, medium and high, and control vs STE). The function “heatmap.2” in R package “gplots” was used. The Ward’s minimum variance method, which aims at finding compact clusters, was used to perform hierarchical clustering. IPA (Qiagen) was used to conduct core analyses of genes that were differentially regulated by medium or high dose WS-CM. A comparison of these core analyses was performed, and activation *Z*-scores were obtained from the Diseases and Functions as well as the Upstream Regulators databases.

### RT-qPCR

Real-time qPCR was carried out using TaqMan™ (Thermo Fisher Scientific) gene expression assays tagged with either FAMMGB or VIC-MGB probes according to the manufacturer’s protocol. In total, 14 TaqMan™ Gene Expression Assay probes were used, and the catalog numbers were provided in the Supplementary file: four housekeeping genes (beta-2-microglobin [*β2M*], beta-actin [*β*-*actin*], peptidylprolyl isomerase A [*PPIA*], and TATA-box binding protein [*TBP*]) and ten genes (*CSF*-*1*, *CSF*-*1R*, *IFN*-*γ*, *IL*-*10*, *IL*-*17A*, *IL*-*17RA*, *IL*-*2*, *IL*-*4*, *IL*-*6*, and *TNF*-*α*). TaqMan™ gene expression assays were selected, based on TaqMan™ recommendation for best coverage of each gene. All TaqMan™ assays were supplied as a 20x stock and used at a final concentration of 1×. The following TaqMan™ probes were tagged with FAM*: β2M*, *β*-*actin*, *IL*-*10*, *IFN*-*γ*, *IL*-*17RA*, *CSF*-*1*, *IL*-*2*. In turn, the following TaqMan™ probes were tagged with VIC: *PPIA*, *TBP*, *IL*-*6*, *TNF*-*α*, *IL*-*17A*, *CSF*-*1R*, *and IL*-*4*. To minimize the number of reactions necessary to conduct the experiment, TaqMan™ probes were multiplexed according to the following (VIC-probe/FAM-probe): *β2M/PPIA*, *β*-*actin/TBP*, *IL10/IL6*, *IFN*-*γ/TNF*-*α*, *IL*-*17RA/IL*-*17A*, *CSF*-*1/CSF*-*1R*, *and IL2/IL*-*4*.

### Cytokine secretion assay

For cytokine analysis, PBMCs were pre-treated with WS-CM or STE and then stimulated with PMA (50 ng/mL), ionomycin (500 ng/mL), and LPS (1 μg/mL) as mentioned above. After 24 hours of stimulation, supernatants were collected and cytokine concentrations (IL-2, IL-4, IL-6, IL-10, TNF-α, IFN-γ, and IL-17A) were measured using a multiplex cytometric bead array assay (Th1/Th2/Th17 Cytokine Kit, BD Biosciences, San Jose, CA) and flow cytometry according to the manufacturer’s instructions. The limits of detection for the cytokines, per the manufacturer, are as follows: IFNγ (3.7 pg/mL), TNF (3.8 pg/mL) and IL-2 (2.6 pg/mL).

## Supplementary information

Supplementary Material

## References

[1] Bagaitkar J, Demuth DR, Scott DA (2008). Tobacco use increases susceptibility to bacterial infection. Tob. Induc. Dis..

[2] Bullen C (2008). Impact of tobacco smoking and smoking cessation on cardiovascular risk and disease. Expert Rev. Cardiovasc. Ther..

[3] Feng Y (2011). Exposure to cigarette smoke inhibits the pulmonary T-cell response to influenza virus and Mycobacterium tuberculosis. Infect. Immun..

[4] Obeidat ME (2018). The genetics of smoking in individuals with chronic obstructive pulmonary disease. Respir. Res..

[5] Sopori M (2002). Effects of cigarette smoke on the immune system. Nat. Rev. Immunol..

[6] Qiu F (2017). Impacts of cigarette smoking on immune responsiveness: Up and down or upside down?. Oncotarget.

[7] Shiels MS (2014). Cigarette Smoking and Variations in Systemic Immune and Inflammation Markers. J. Natl. Cancer Inst..

[8] Forsslund H (2014). Distribution of T-Cell Subsets in BAL Fluid of Patients With Mild to Moderate COPD Depends on Current Smoking Status and Not Airway Obstruction. Chest.

[9] Stampfli MR, Anderson GP (2009). How cigarette smoke skews immune responses to promote infection, lung disease and cancer. Nat. Rev. Immunol..

[10] Arcavi L, Benowitz NL (2004). Cigarette smoking and infection. Arch. Intern. Med..

[11] Zeller M, Hatsukami D (2009). The Strategic Dialogue on Tobacco Harm Reduction: a vision and blueprint for action in the US. Tob. Control.

[12] Rom O, Avezov K, Aizenbud D, Reznick AZ (2013). Cigarette smoking and inflammation revisited. Respir. Physiol. Neurobiol..

[13] Arimilli S, Madahian B, Chen P, Marano K, Prasad GL (2017). Gene expression profiles associated with cigarette smoking and moist snuff consumption. BMC Genomics.

[14] Arimilli Subhashini, Schmidt Eckhardt, Damratoski Brad E., Prasad G. L. (2017). Role of Oxidative Stress in the Suppression of Immune Responses in Peripheral Blood Mononuclear Cells Exposed to Combustible Tobacco Product Preparation. Inflammation.

[15] Mian MF, Lauzon NM, Stämpfli MR, Mossman KL, Ashkar AA (2008). Impairment of human NK cell cytotoxic activity and cytokine release by cigarette smoke. J. Leukoc. Biol..

[16] Mian MF, Pek EA, Mossman KL, Stampfli MR, Ashkar AA (2009). Exposure to cigarette smoke suppresses IL-15 generation and its regulatory NK cell functions in poly I:C-augmented human PBMCs. Mol. Immunol..

[17] Kalra R, Singh SP, Savage SM, Finch GL, Sopori ML (2000). Effects of cigarette smoke on immune response: chronic exposure to cigarette smoke impairs antigen-mediated signaling in T cells and depletes IP3-sensitive Ca(2+) stores. J. Pharmacol. Exp. Ther..

[18] Metcalfe HJ (2014). Effects of cigarette smoke on Toll-like receptor (TLR) activation of chronic obstructive pulmonary disease (COPD) macrophages. Clin. Exp. Immunol..

[19] Manzel LJ, Shi L, O’Shaughnessy PT, Thorne PS, Look DC (2011). Inhibition by cigarette smoke of nuclear factor-kappaB-dependent response to bacteria in the airway. Am. J. Respir. Cell Mol. Biol..

[20] van Leeuwen DM (2005). Differential Gene Expression in Human Peripheral Blood Mononuclear Cells Induced by Cigarette Smoke and Its Constituents. Toxicol. Sci..

[21] Arimilli S, Makena P, Liu G, Prasad GL (2019). Distinct gene expression changes in human peripheral blood mononuclear cells treated with different tobacco product preparations. Toxicol. In Vitro.

[22] Mertens Tinne C. J., van der Does Anne M., Kistemaker Loes E., Ninaber Dennis K., Taube Christian, Hiemstra Pieter S. (2017). Cigarette smoke differentially affects IL-13-induced gene expression in human airway epithelial cells. Physiological Reports.

[23] Chen H, Cowan MJ, Hasday JD, Vogel SN, Medvedev AE (2007). Tobacco smoking inhibits expression of proinflammatory cytokines and activation of IL-1R-associated kinase, p38, and NF-kappaB in alveolar macrophages stimulated with TLR2 and TLR4 agonists. J. Immunol..

[24] Ouyang Y (2000). Suppression of human IL-1beta, IL-2, IFN-gamma, and TNF-alpha production by cigarette smoke extracts. J. Allergy Clin. Immunol..

[25] Pelham CJ, Agrawal DK (2014). Emerging roles for triggering receptor expressed on myeloid cells receptor family signaling in inflammatory diseases. Expert Rev. Clin. Immunol..

[26] Palumbo R (2007). Cells migrating to sites of tissue damage in response to the danger signal HMGB1 require NF-kappaB activation. J. Cell Biol..

[27] Cheng Y (2017). HMGB1 translocation and release mediate cigarette smoke-induced pulmonary inflammation in mice through a TLR4/MyD88-dependent signaling pathway. Mol. Biol. Cell.

[28] Georgescu M-M (2010). PTEN Tumor Suppressor Network in PI3K-Akt Pathway Control. Genes Cancer.

[29] Moraes LA, Piqueras L, Bishop-Bailey D (2006). Peroxisome proliferator-activated receptors and inflammation. Pharmacol. Ther..

[30] Cuzzocrea S (2004). Role of endogenous and exogenous ligands for the peroxisome proliferators activated receptors alpha (PPAR-alpha) in the development of inflammatory bowel disease in mice. Lab. Invest..

[31] Shen W (2014). Negatively regulating TLR4/NF-κB signaling via PPARα in endotoxin-induced uveitis. Biochim. Biophys. Acta. Mol. Basis Dis..

[32] Arimilli S, Damratoski BE, Bombick B, Borgerding MF, Prasad GL (2012). Evaluation of cytotoxicity of different tobacco product preparations. Regul. Toxicol. Pharmacol..

[33] Arimilli S, Damratoski BE, Prasad GL (2013). Combustible and non-combustible tobacco product preparations differentially regulate human peripheral blood mononuclear cell functions. Toxicol. In Vitro.

[34] Arimilli, S., Damratoski, B. E. & Prasad, G. L. Methods to evaluate cytotoxicity and immunosuppression of combustible tobacco product preparations. *J. Vis. Exp*., 52351, 10.3791/52351 (2015). 10.3791/52351 PMC4354515 25650834

[35] Cawood TJ, Moriarty P, O’Farrelly C, O’Shea D (2007). Smoking and thyroid-associated ophthalmopathy: A novel explanation of the biological link. J. Clin. Endocrinol. Metab..

