# Supplementary material for: Cigarette smoke preparations, not moist snuff, impair expression of genes involved in immune signaling and cytolytic functions
Source: Sci Rep. 2019 Sep 16;9:13390. doi: 10.1038/s41598-019-48822-w (PMC6746724; doi:10.1038/s41598-019-48822-w)
Supplement: Supplementary file 1 — Supplementary Material [file 41598_2019_48822_MOESM1_ESM.docx]

**Supplementary Information**

# **Cigarette smoke preparations, not moist snuff, impair expression of genes involved in immune signaling and cytolytic functions**

SREP-19-11526

Gang Liu^1^, Subhashini Arimilli^2^, Evan Savage^3^, and G. L. Prasad^1^

^1^RAI Services Company, Winston Salem, NC, 27105, United States

^2^Eurofins Lancaster Laboratories PSS, Winston Salem, NC 27105, United States

^3^Genome Explorations, Memphis, TN 38132, United States

**Supplementary Table S1** – Summary list of all significantly differentially expressed probesets when PBMCs were treated with medium WS-CM (1 µg/ml equal-nicotine units). Column headers are: ProbeID: Probe identification number, Gene, Gene Name, p_val_unAdjust: raw p value computed using ANOVA, Pval_adj_FDR0.01, FDR-adjusted p value (0.01 as cut-off), FC, fold change.

**Supplementary Table S2** – Summary list of all significantly differentially expressed probe sets and their corresponding genes when PBMCs were treated with high WS-CM (3 µg/ml equal-nicotine units). Column header descriptions were provided in Supplementary Table S1.

**Supplementary Table S3** – Pearson Correlation of select gene expression levels measured by microarray and qRT-PCR experiments. The expression of the following genes was assessed by qRT-PCR: CSF1, CSF1R, IFNG, IL10, IL17A, IL17RA, IL2, IL4, IL6, TNF.

**Supplementary Table S4 –** A list of z-scores describing the impact of medium and high WS-CM treatments on curated biological pathways. The first column lists all the canonical pathways in IPA knowledge database, the second and third columns provide the corresponding z-scores for medium and high WS-CM treatments.

**Supplementary Table S5** – A list of z-scores describing the impact of medium and high WS-CM treatments on upstream regulators. The fist column lists all the upstream regulators in IPA knowledge database, the second and third columns are the corresponding z-scores in the condition of medium and high WS-CM treatment.

**Supplementary Table S6 –** A list of z-scores describing the impact of medium and high WS-CM treatment on disease pathways and biological functions. The fist column lists all the upstream regulators in IPA knowledge database, the second and third columns are the corresponding z-scores in the condition of medium and high WS-CM treatment.

## Supplementary Figure S1: Comparison of gene expression in PBMCs treated with medium and high doses of WS-CM. (a) Venn diagram of differentially expressed genes in PBMCs that were up-regulated (>2-fold change) following treatment with high or medium doses of WS-CM. (b) Venn diagram of differentially expressed genes in PBMCs that were down-regulated (>2-fold change) following treatment with high or medium doses of WS-CM. A notable number of genes were commonly upregulated (278) and downregulated (806) between the medium and high doses of WS-CM.

# **Supplementary Figure S1.**


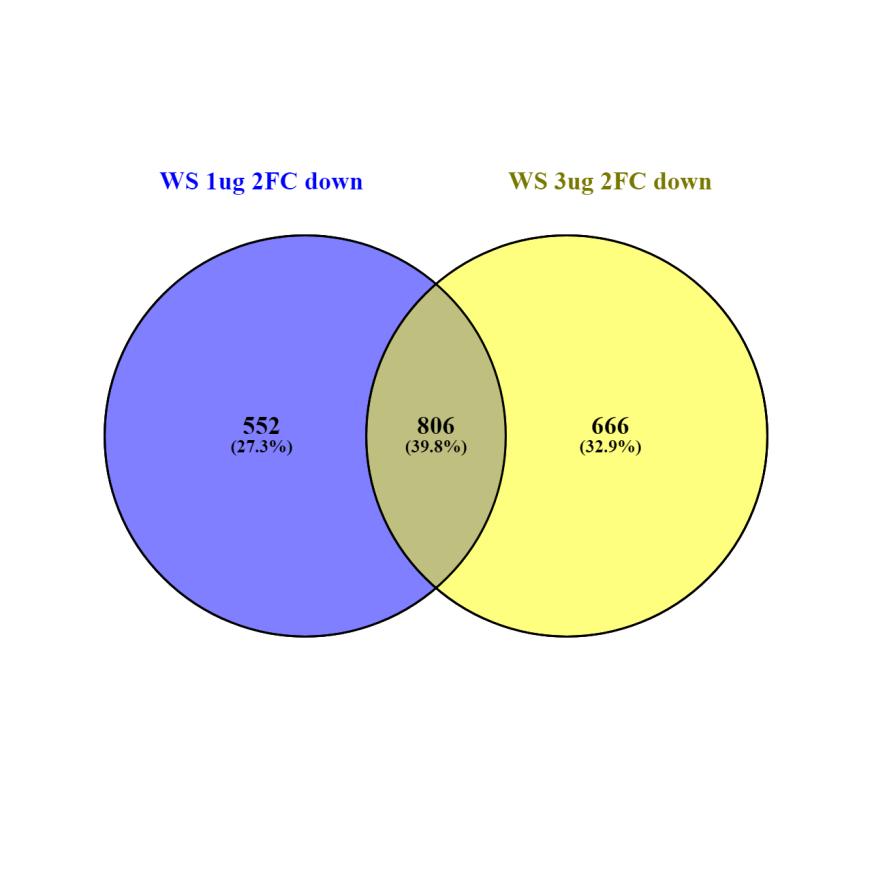


High WS-CM

Medium WS-CM


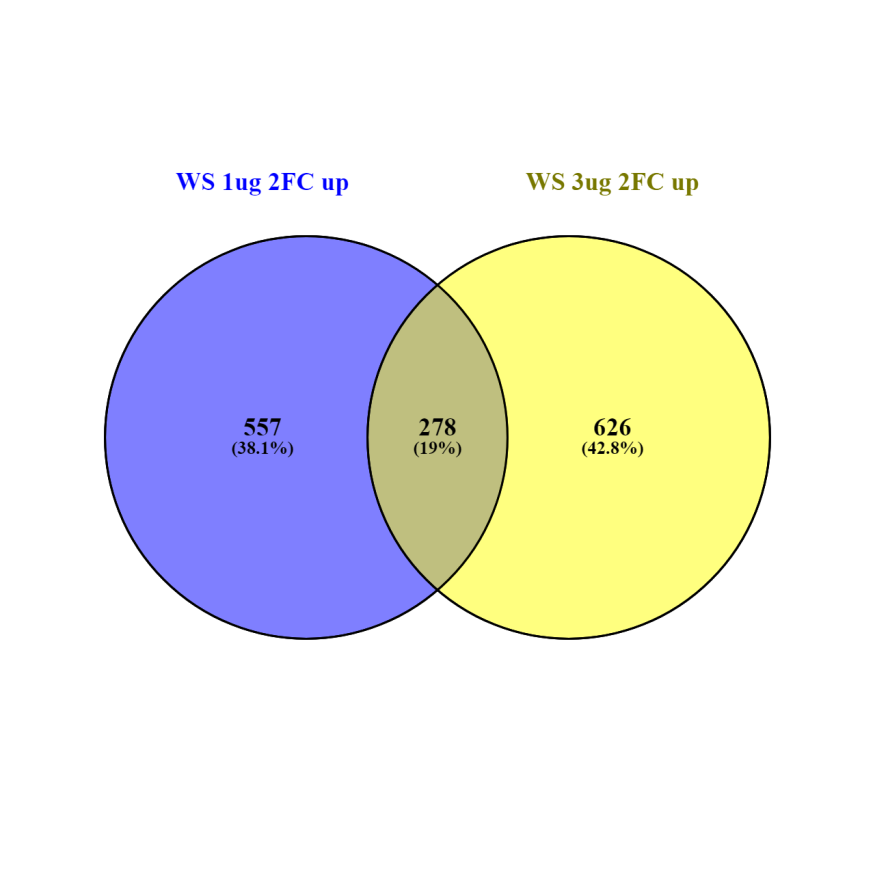


Medium WS-CM

High WS-CM

A

B
